# The Use of Humic Substances as an Additive to Feed Mixtures in Pheasant Breeding

**DOI:** 10.3390/ani15152321

**Published:** 2025-08-07

**Authors:** Alena Hreško Šamudovská, Stanislav Hreško, Iveta Maskaľová, Alica Tvrdá, Lukáš Bujňák

**Affiliations:** Department of Animal Nutrition and Husbandry, University of Veterinary Medicine and Pharmacy in Košice, Komenského 73, 041 81 Košice, Slovakia; alena.hreskosamudovska@uvlf.sk (A.H.Š.); iveta.maskalova@uvlf.sk (I.M.); alica.tvrda@student.uvlf.sk (A.T.); lukas.bujnak@uvlf.sk (L.B.)

**Keywords:** pheasant, feed, natural feed additives, humic substances, growth performance, digestive organs, biochemical parameters, short-chain fatty acids, excretion

## Abstract

The effect of humic substances on growth performance, excretion, and the digestive organs was studied in pheasant chickens. Three hundred 1-day-old pheasant chicks were assigned to two equal groups. Chicks in the experimental group were fed diets supplemented with humic substances (5 g/kg). Chickens in the experimental group reached significantly higher average daily weight gains (*p* ˂ 0.05) in the second phase of rearing (29–49 d) at a significantly better feed conversion ratio (*p* ˂ 0.05). The supplementation of humic substances led to a significant increase in the average body weight of pheasants at the end of the experiment (*p* ˂ 0.05), and to a significant increase in average daily weight gains for the entire monitored period (*p* ˂ 0.05). In the experimental group, significantly higher relative weights of the gizzard (*p* ˂ 0.05), small intestine (*p* ˂ 0.05), and jejunum (*p* ˂ 0.05), along with a significantly larger relative length of the duodenum (*p* ˂ 0.05), were recorded. A significantly higher concentration of dry matter in the droppings of pheasants was found in the experimental group than in the control group (*p* ˂ 0.05). According to our results, feeding humic substances had the most beneficial effect on pheasant growth performance during the growing phase. Humic substances can also have a positive effect on the microclimate within the breeding facility. Reductions in the water content of droppings contribute to a reduction in the humidity of the litter, thus limiting the activity of nitrogenic bacteria.

## Simple Summary

Various feed supplements can be used to influence production and health parameters in poultry farming, including pheasant farming. Humic substances are interesting compounds of bioactive matter that have found valuable application in agriculture, industry, medicine, and pharmacology. The addition of humic substances to feed or water has been shown to positively influence production parameters in different farm animal species. In poultry, improvements in growth performance, feed intake, feed conversion ratio, egg production, carcass yields, the quality of meat, and biochemical parameters have been demonstrated. The aim of this study was to investigate the effect of supplementing feed mixtures with humic substances based on leonardite (Humac s.r.o., Košice, Slovakia) on growth performance, nutrient excretion, the weight of the digestive organs, the length of small intestine segments, the fermentation activity of the microbial population in the lumen of the digestive tract, and some biochemical parameters in pheasant chickens (*Phasianus colchicus*). The results of our study show that the addition of humic substances to feed mixtures in the amount of 5 g per kg can have a positive effect on the growth performance of pheasant chickens in the second phase of rearing.

## 1. Introduction

Pheasants are attractive and interesting birds that have become increasingly popular in domestic breeding in recent years. These beautiful birds come from Asia, mainly from the southeast region. There are many species of pheasant, of which the common pheasant (*Phasianus colchicus*), also known as the ring-necked pheasant or hunting pheasant, is perhaps the most widespread. They have been introduced and spread in many parts of the world, including Europe and North America. Other popular species for domestic breeding include Reeves’s pheasant (*Syrmaticus reevesii*), the silver pheasant (*Lophura nycthemera*), the golden pheasant (*Chrysolophus pictus*), also known as the Chinese pheasant or rainbow pheasant, and Lady Amherst’s pheasant (*Chrysolophus amherstiae*), also known as the diamond pheasant. Pheasant breeding is practiced for several reasons. First, pheasant meat is very tasty and nutritious, as well as being high in protein and low in fat [1,2]. Pheasant eggs are also edible. Secondly, pheasants are often kept as luxurious decorations in gardens, parks, or private plots, since many species have a unique appearance with beautiful variegated and shiny feathers. Third, pheasant breeding can be part of state programs aimed at protecting and restoring wild pheasant populations, as the numbers of individuals in wild pheasant populations have declined in recent decades [3]. Finally, pheasants, mainly common pheasants, are traditionally used for hunting and are often kept for this purpose in special pheasant pens.

The breeding of gamebirds became more intensive due to growing popularity and higher demand from hunters. So the gamebird farming approached the methods of domestic poultry farming. Somehow, the idea came naturally to positively influence the production of pheasants through the use of various feed supplements, in a way that can be practiced in large-scale poultry farms. Antibiotics have been used as growth stimulants in the past. However, their use in animal nutrition was banned in Europe in 2006 as a response to the increasing problem of antibiotic resistance [4]. Among the potential bioactive substances that could positively affect animal health and improve production indicators, there is no doubt that humic substances—the use of which has attracted considerable interest over the last two decades in the field of animal nutrition —are also included. Humic substances comprise a group of natural organic compounds formed in the soil. They mainly consist of fractions of humic acids, fulvic acids, and humics; to a lesser extent, they contain ulmic acids and trace amounts of minerals such as iron, manganese, copper, and zinc [5,6]. They have found valuable applications in agriculture and industry, but also in the field of environmental protection. They are used in human and veterinary medicine and in pharmacology for their analgesic, antimicrobial, and anti-inflammatory effects [7].

The addition of humic substances to feed or water has been shown to positively influence the production parameters of different farm animal species. For example, pigs that were fed diets supplemented with humic substances showed higher average body weight gains, a better feed conversion ratio, and reduced ammonia excretion [8]. In another study, humic substances also affected the blood count, hematocrit, hemoglobin, cholesterol, mineral levels, and biomarkers of oxidative stress in piglets. In addition, the diversity of microbiota in feces also changed, which could contribute to the better utilization of nutrients from feed [9]. Production and blood parameters in rabbits were similarly affected [10,11]. Even in ruminants, the intake of humic substances in the diet had an effect on some parameters. In dairy cows, the nutrient intake, rumen fermentation profile, biochemical parameters, milk yield, and fatty acid profile were positively affected [12]. In goats, in addition to increased milk yield, there was also a significant reduction in blood cholesterol and blood serum LDL cholesterol [13].

In poultry, the addition of humic substances to the diets or water was shown to improve growth performance, feed intake, feed conversion ratio, egg production, carcass yields, quality of meat, and biochemical parameters [14,15,16,17]. The results of more detailed studies indicate that humic substances could also influence not only the intestinal microflora and immunity, but also the height of the villi and the depth of the crypts of the small intestine [18,19,20]. In turkeys, in addition to monitoring production parameters, blood parameters, and yield, humic acids were also tested to evaluate their effectiveness as an absorbent for aflatoxin B1 with a positive result [21]. Only a few studies using humic substances have been carried out on pheasants, mostly with positive results. The supplementation of humic substances to the diet of farm pheasants increased the carcass yields and influenced the quality of meat, as shown in the work of Gálik et al. [22]. Humic substances demonstrably affected the metabolic and biochemical parameters in the blood of pheasants, as well as the parameters of the antioxidant enzymatic system [23,24].

The aim of this study was to investigate the effect of supplementing feed mixtures with humic substances on growth performance, nutrient excretion, the weight of the digestive organs, the length of individual segments of the small intestine, the fermentation activity of the microbial population in the lumen of the digestive tract, and some biochemical parameters in pheasant chickens.

## 2. Materials and Methods

### 2.1. Animals and Experimental Design

For the experiment, three hundred one-day-old pheasant chicks (*Phasianus colchicus* L.) with a starting average body weight of 18.48 g were used, which were randomly divided into two groups (n = 150; five replicate pens per group) and placed on deep litter in premises meeting the required zootechnical conditions. The experimental period lasted 49 days. During the experiment, the pheasants were fed complete feed mixtures based on corn, wheat, soybean meal, and fish meal according to the growth phases (starter phase 1–28 days, growth phase 29–49 days). Pheasants in the experimental group were fed mixtures with the addition of humic substances based on leonardite (Humac s.r.o., Košice, Slovakia) in the amount of 5 g per kg of feed mixture according to the manufacturer’s recommendation. Characteristics of the preparation: particle size up to 100 µm, pH 5.8, moisture max. 15%, humic acids in dry matter min. 65%, free humic acids in dry matter min. 60%, fulvic acids in dry matter min. 5%, Ca 42,278 mg/kg, Mg 5111 mg/kg, Fe 19,046 mg/kg, Cu 15 mg/kg, Zn 37 mg/kg, Mn 142 mg/kg, Co 1.24 mg/kg, Se 1.67 mg/kg, V 42.1 mg/kg, and Mo 2.7 mg/kg. Feed and water intake were provided ad libitum.

On the 1st, 28th, and 49th days of the experiment, both the weight of pheasant chicks and feed consumption were recorded. The feed conversion ratio was expressed as feed consumption per kilogram of gain. During the 42nd and 43rd days of the experiment, samples of pheasants’ droppings were taken to determine the excretion of dry matter and crude protein. Droppings were collected from random chickens immediately after excretion onto a clean, solid pad to avoid any contamination. Ten samples were analyzed for each group. Samples were a mixture of droppings from several birds.

At the end of the experiment (49th day), 10 pheasants were randomly selected from each group, from which blood samples were taken from the jugular vein. Subsequently, the birds were stunned with a percussive blow to the head and killed by exsanguination. During the following evisceration, the digestive organs (proventriculus, gizzard, small intestine, cecum, pancreas, and liver) were separated, including individual segments of the small intestine: duodenum (from the pyloric junction to the distalmost point of insertion of the duodenal mesentery), jejunum (from the distalmost point of insertion of the duodenal mesentery to the junction with Meckel’s diverticulum), and ileum (from the junction with Meckel’s diverticulum to the ileocecal junction). All digestive organs and individual parts of the intestine were weighed, and the length of small intestine segments (duodenum, jejunum, and ileum) was measured. The relative weight of digestive organs and the relative length of small intestine segments were expressed as a portion of body weight (g or cm/100 g body weight). The total weight and length of the small intestine were calculated as the sum of the weights and lengths of the individual segments of the small intestine, respectively. The cecums were separated in order to evaluate the fermentation activity of the microbial population in the lumen of the digestive tract by determining the content of short-chain fatty acids and pH in the contents of the cecums.

The housing of the animals and all handling of the animals during the experiment were carried out in accordance with the requirements for the protection of animals used for scientific or educational purposes established by Government Regulation No. 377/2012 and the decree of the Ministry of Agriculture and Rural Development of the Slovak Republic No. 436/2012 implementing Directive 2010/63/EU of the European Parliament and the Council on the protection of animals used for scientific purposes [25].

### 2.2. Laboratory Analysis

The content of nutrients in the used feed mixtures (dry matter, crude protein, ether extract, crude fiber, ash) was determined by analytical methods according to EC Commission Regulation 152/2009 [26]. The analyzed content of nutrients and metabolizable energy in the feed mixtures used in individual rearing phases is shown in Table 1.

Fresh samples of pheasants’ droppings from each collection were thoroughly homogenized, weighed, and dried in a hot air oven at 60 °C to a constant weight for the determination of dry matter content. Subsequently, after weighing, the dried droppings samples were finely ground for the analysis of crude protein using the Kjeldahl procedure.

The concentration of short-chain fatty acids (acetic, propionic, butyric acid) in the contents of the cecum was analyzed by a two-capillary isotachophoretic analyzer (EA100, VILLA LABECO, Slovak Republic). The supernatant obtained by diluting 2 g of fresh cecal content with distilled water (1:100) and subsequent centrifugation was used for analysis.

The pH of cecal contents was measured by placing an electrode (pH electrode XS Sensor 2-Pore T; Giorgio Bormac, Italy) directly into the contents.

Blood serum was used for the analysis of selected metabolites and minerals in the blood, which was obtained by centrifugation of clotted blood (3000 rpm for 30 min). Total protein, albumin, urea, uric acid, glucose, total lipids, cholesterol, triacylglycerols, alkaline phosphatase (ALP), aspartate aminotransferase (AST), calcium, and phosphorus values were determined in blood serum using a fully automatic random-access benchtop analyzer (Ellipse, Italy). Finally, the concentration of globulin was calculated by subtracting albumin values from total protein values.

### 2.3. Statistical Analysis

The obtained results were statistically evaluated using an unpaired t-test using GraphPad Prism 8.0 statistical software (GraphPad Software, San Diego, CA, USA). Differences were considered significant at a level of *p* < 0.05. Average body weight, daily weight gain, daily feed intake, and feed conversion ratio were evaluated for each pen (five replicate pens per group). Expression of results: mean value ± standard error of the mean (SEM).

## 3. Results

### 3.1. Growth Performance

Data on the average live body weight of pheasant chicks during the experiment are shown in Table 2. At the end of the experiment (day 49), a significantly higher average live body weight of birds was recorded in the experimental group than in the control group (*p* ˂ 0.05).

The average daily weight gains in individual rearing phases are shown in Table 3. While in the first phase of rearing (1–28 days), slightly higher average daily weight gains were recorded in the control group than in the experimental group; in the second phase of rearing (29–49 days), significantly higher average daily weight gains were recorded in the experimental group than in the control group (*p* ˂ 0.05). Average daily weight gains of pheasant chicks were also significantly higher in the experimental group for the entire monitored period (*p* ˂ 0.05).

Comparing the control to the experimental group, the average daily feed intake was not affected by the addition of humic substances to the feed in both rearing phases, as well as for the entire monitored period (Table 3).

Evaluation of feed conversion ratio in the individual stages of rearing is shown in Table 3. In the experimental group, in the first stage of rearing, the value of this indicator was found to be significantly higher (*p* ˂ 0.05), and in the second stage of rearing, it was significantly lower (*p* ˂ 0.05) than in the control group. For the entire monitored period, the feed conversion ratio was comparable in both groups.

### 3.2. Examination of Pheasant Droppings

**Figure 1:**
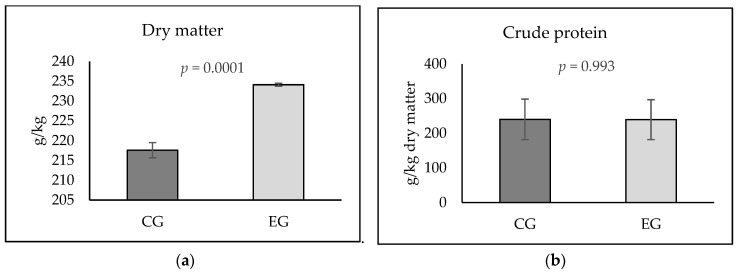
The influence of humic substances on (**a**) the content of dry matter and (**b**) crude protein in pheasant droppings. CG—control group; EG—experimental group. Significant difference: *p* ˂ 0.05.

The results of the droppings examination are shown in Figure 1. A significantly higher dry matter content was found in the droppings of pheasant chickens of the experimental group (234.13 ± 0.36 g/kg) than in the droppings of the control group (217.60 ± 1.93 g/kg; *p* ˂ 0.05). The content of crude protein in the droppings of pheasant chickens was not significantly affected by the addition of humic substances to the feed.

### 3.3. Digestive Organs Characteristics

In the group with the addition of humic substances, compared to the control group, a significantly higher relative weight of the gizzard (*p* ˂ 0.05) and small intestine (*p* ˂ 0.05) was recorded (Table 4). The relative weight of the proventriculus, pancreas, and liver was not statistically significantly affected.

When evaluating the individual segments of the small intestine, a significantly higher relative weight of the jejunum was recorded in the experimental group (*p* ˂ 0.05) than in the control group (Table 4). Likewise, the relative weight of the duodenum and ileum was higher in the experimental group than in the control group, but the differences were not statistically significant.

In the experimental group, a statistically significantly greater relative length of the duodenum (*p* ˂ 0.05) was found, as well as a statistically significantly greater percentage of the duodenum in the total length of the small intestine (*p* ˂ 0.05) than in the control group (Table 5). The relative length of the jejunum, ileum, and the whole small intestine were also bigger in the experimental group, but the differences were not significant in comparison to the control group.

### 3.4. Short-Chain Fatty Acids and pH in the Contents of the Cecum

The fermentation activity of the microbial population in the lumen of the digestive tract was evaluated by analyzing the concentration of short-chain fatty acids (acetic, propionic, and butyric acids) and measuring pH in the contents of the cecum. The results of this analysis are presented in Table 6. The addition of humic substances to the feed did not have a significant effect on the concentration of acetic, propionic, and butyric acids, as well as on the total content of short-chain fatty acids in the cecum contents of pheasants. Likewise, the pH of the cecum contents was not statistically significantly affected.

### 3.5. Serum Biochemical Parameters

The results of the biochemical examination of the blood serum are summarized in Table 7. By statistical analysis, a significant difference between the groups was noted only for the calcium level, which was lower in the experimental group than in the control group (*p* ˂ 0.05). The other monitored indicators were not statistically significantly affected by the addition of humic substances to the feed.

## 4. Discussion

The results of the monitored production parameters in our experiment demonstrate that the addition of humic substances to the feed can have a positive effect on the growth of pheasant chickens. However, the beneficial effect only became apparent in the second phase of rearing. In the group with the addition of humic substances, significantly higher weight gains were recorded in the growth phase at a significantly better feed conversion ratio than in the control group. In the first phase of rearing, weight gains were not significantly affected, but a significantly worse feed conversion ratio was found in the experimental group. Likewise, for the entire observed period, significantly higher average daily weight gains were found in the experimental group, which was manifested by a significant increase in the live weight of the birds at the end of the experiment. A significant increase in the live weight of pheasants after the addition of humic substances to the feed (in 0.5 and 0.75% concentration) was also noted by Gálik et al. [22]. A positive effect on growth in the growth phase, as in our study, was observed by Ozturk et al. [15], who observed the effect of adding different doses of humic substances to feed mixtures (0.5, 1.0, 1.5 g/kg) in broiler chickens. In the chickens that were fed with a diet containing humic substances in the amount of 1.5 g/kg, significantly higher weight gains were recorded in the growth phase of fattening as well as during the entire monitored period at a significantly better feed conversion ratio than in the group without the addition of humic substances. Similar results were reported by Kocabağli et al. [27] in broiler chickens that received a diet enriched with humic substances in the amount of 2.5 kg/tonne during the entire experimental period. While chicken body weight and feed conversion ratio after 21 days of the experiment were not affected, the chickens in the group with the addition of humic substances achieved on day 42 numerically higher weights in comparison to the control group. Moreover, the feed conversion ratio was significantly better in the experimental group for the period from day 22 to 42. The addition of humic substances, similar to our study, did not have a significant effect on the feed conversion ratio over the entire monitored period.

Our results are partially in agreement with the results of the study by Avci et al. [28], who observed the effect of adding different doses of humic acids to feed mixtures (360, 480, 600 mg/kg) in quails. In the first phase of the experiment, the addition of humic acids led to a decrease in weight gain and a deterioration of the feed conversion ratio. The beneficial effect of the monitored substances was manifested only in the growth phase, in which the quails achieved higher weight gains at a better feed conversion ratio. For the entire observed period, no statistically significant differences were recorded in the monitored production parameters compared to the control group.

Significantly higher final body weight, higher weight gains, and significantly better feed conversion ratio over the entire observation period were recorded in Japanese quails receiving diets supplemented with humic substances in amounts of 10, 20, and 30 mL/kg [29]. Similar results were also reported in broiler chickens fed diets containing humic acids at 0.1, 0.2, or 0.3% concentrations [18,30] and chickens fed diets supplemented with humic acids in amounts of 1.5, 2.25, and 3 g/kg [16]. Arif et al. [16] report that the beneficial effect of the administration of humic acids in the amounts of 2.25 and 3 g/kg was already manifested in the starter phase of fattening chickens. Similar results were found in Sasso chickens [31].

In some studies [18,32,33,34], as well as in our experiment, feed intake was not significantly affected by the administration of humic substances. However, several researchers report a significant reduced feed intake in poultry after the administration of humic substances while achieving comparable or higher weight gains than in the group without the addition of humic substances [15,16,29,31,35,36].

The results of various studies show that the improvement of production parameters in poultry after the administration of humic substances occurs as a result of an increase in the utilization of nutrients from feed, which is indicated by an improvement in feed conversion ratio. Higher utilization of nutrients from feed can be mediated, for example, by increased activity of digestive enzymes. Mao [33], who studied fulvic acids, concluded that the above-mentioned acids can induce the expression of intestinal digestive enzymes. In the chyme of the intestinal tract of broiler chickens fed with a diet enriched with fulvic acids (in the amount of 0.2, 0.6, and 1 g/kg), a significantly higher activity of protease, lipase, and amylase was found compared to the control group. In all of these experimental groups, compared to the control group, significantly higher final body weight of chickens, significantly higher weight gains, and better feed conversion ratio (significant at medium concentration) were recorded. An increase in the activity of digestive enzymes in broiler chickens was also recorded after the addition of humic acids to drinking water [32].

Also, in some studies on poultry, due to the administration of humic substances, an increased digestibility of feed nutrients (crude protein, ether extract, or crude fiber) was recorded [30,31,37]. An increase in the digestibility of crude protein also leads to a decrease in their excretion and subsequently to a decrease in the formation of volatile ammonia. Volatile ammonia is the result of microbial transformation of nitrogenous substances found in poultry litter, and its high concentrations in the air of breeding areas negatively affect the health and productivity of animals [38]. However, in the current study, no significant reduction in the excretion of crude protein due to the addition of humic substances to the feed was observed. On the other hand, a significantly higher dry matter content was found in the droppings of the experimental group than in the droppings of the control group. A higher dry matter content in droppings can also contribute to improving the microclimate, as a lower water content in litter limits microbial activity [39]. We recorded similar results in our earlier study in broiler chickens [40]. In the droppings of chickens that were fed feed enriched with humic substances (in an amount of 5 g/kg in the starter diet and 7 g/kg in the grower and finisher diet), the analysis revealed a significantly higher dry matter content than in the droppings of the control group. However, in addition to the increase in dry matter content, a significant decrease in the content of crude protein was also recorded, which indicates that the addition of humic substances can lead to an increase in the utilization of nitrogenous substances from the feed.

An increase in the relative weight of digestive organs, as well as an increase in the relative length of the small intestine, could be considered an indication of better digestion and utilization of nutrients. In the present study, a significantly higher relative weight of the gizzard, entire small intestine, and jejunum was recorded in the group of pheasants with the addition of humic substances. Also, a significantly larger relative length of the duodenum was found in this group.

A larger, well-developed gizzard can serve more effectively as a mixing space for ingested feed with digestive juices, which can lead to improved nutrient digestibility [41]. Moreover, increased release of cholecystokinin can occur, which stimulates the secretion of pancreatic juice rich in digestive enzymes [42]. Individual segments of the small intestine, together with the pancreas and proventriculus, are the main organs producing digestive enzymes in poultry. Increased length of the intestine can be associated with a reduced rate of passage of intestinal contents, together with prolonged enzymatic digestion This can contribute to improving the digestibility of nutrients [42,43].

A higher gizzard weight in pheasants due to the addition of humic substances to the feed was also noted by Gálik et al. [22]. Similar results were also found in Sasso chickens, which received a diet enriched with humic acids in a 0.2% concentration. However, the addition of a higher concentration of humic acids (0.4%) led to a decrease in the relative weight of the gizzard but, on the other hand, an increase in the relative weight of the intestine compared to the control group. The relative length of the intestine was significantly lower in all groups of chickens that were given humic acids than in the group without treatment [31]. Arif et al. [35], who studied humic acids in quail, did not note the effect of the monitored substances on gizzard weight, but the addition of the highest tested concentration of humic acids (2.25 g/kg diet) led to an increase in the weight and length of the intestine. Higher intestine weight was also recorded when humic acids were administered in the amount of 1.5 g/kg feed.

Taklimi et al. [18], Abdel-Mageed [29], Lala et al. [32], and Abdl Razek et al. [10] also studied the influence of humic substances on intestinal morphology. They found that the addition of humic substances can lead to an increase in the villi of the intestinal mucosa. Taller villi represent a larger absorption surface. This is associated with a better utilization of nutrients from the feed. Short-chain fatty acids (mainly acetic, propionic, and butyric acids) play an important role in the development of the intestines and the maintenance of the integrity of intestinal epithelial cells. They are the main metabolites of microbial fermentation of undigested carbohydrates, especially in the cecum. Butyric acid has a special physiological significance because this short-chain fatty acid is the primary source of energy for epithelial cells. Through antiperistaltic movements, it can move from the cecum to the small intestine and subsequently support its development [44].

From the results of our analysis of the concentration of short-chain fatty acids in the contents of the cecum, it is clear that the addition of humic substances had no significant effect on the microbial activity in the lumen of the digestive tract of pheasant chickens. Similar results were published in a study by Shermer et al. [45] and in our recent study in turkeys [46]. Also, in our earlier study on broiler chickens, the concentrations of short-chain fatty acids were not significantly affected by the addition of humic substances to the feed (in an amount of 5 g/kg in the starter diet and 7 g/kg in the grower and finisher diet); however, a significant decrease in the pH of the contents of the cecum was detected [40]. Lowering the pH in the digestive tract creates an unsuitable environment for the growth of pathogens. The same effect on the pH of the intestinal content was also recorded in broilers receiving a diet containing humates in the amount of 2.5 kg/tonne [47] and in chickens receiving humic acids (1 or 2 mL/L) through drinking water [48]. However, in our study on pheasants as well as in the study by Mohammadsadeghi et al. [49] on broiler chickens, the addition of humic substances in the concentration used had no significant effect on the pH of the intestinal contents.

Some scientists report in their studies that the administration of humic substances can lead to a significant decrease in the levels of cholesterol, total lipids, or triglycerides or, for example, to an increase in the concentration of glucose and total protein in poultry blood [15,30,31]. In the present study, there was no significant effect of the addition of humic substances to the feed on the monitored biochemical parameters in the blood of pheasant chickens, except for the effect on the calcium level. The concentration of calcium in the blood serum was significantly lower in the experimental group compared to the control group. The decrease in the level of calcium in the blood of pheasant chickens of the experimental group may be, for example, a consequence of the increased accumulation of this mineral element in the bones. This hypothesis is confirmed by the results of the study by Jaďuttová et al. [50], who investigated the influence of humic substances from the same source in broiler chickens. In chickens with the addition of humic substances to the feed in a concentration of 0.8%, a significantly lower concentration of calcium in the blood serum was found, as well as a higher content of calcium in the tibia compared to the group without addition. Similar results were also recorded in a study on Japanese quail [29]. However, studies are also available in which the administration of humic substances led to an increase in the concentration of calcium in the blood of poultry [28], or no effect was recorded [15,23,51].

The discrepancy in the results of the influence of humic substances obtained from various studies can be attributed to the use of humic substances preparations that differ in composition, particularly in the representation of the main fractions of humic substances, such as fulvic acids and humic acids. Additionally, the amount of humic substances administered through feed or water, the duration of administration, and the species of the target animal all contribute to the discrepancy.

## 5. Conclusions

The results of our study show that the addition of humic substances to feed mixtures in the amount of 5 g per kg can have a positive effect on the growth performance of pheasant chickens. However, the positive impact was manifested only in the second phase of rearing. The improvement in growth performance after the administration of humic substances can be a consequence of better digestion and utilization of nutrients from the feed. The positive effect of the administration of humic substances can also be manifested when influencing the microclimate in the breeding facility by reducing the water content in the litter through the excretion of droppings with a higher dry matter content. Since the effect of humic substances was only evident in the growth phase of rearing, the question is the duration of their administration. The determination of the optimal concentration of humic substances in the diet of pheasants also remains questionable. Therefore, and also to clarify the hypotheses regarding the mechanism of action, further studies are needed.

## Figures and Tables

**Table 1:** Nutrient and energy content in feed mixtures used in the experiment.

	Starter Feed Mixtures (1–28 Days)		Grower Feed Mixtures (29–49 Days)	
	CG	EG	CG	EG
Dry matter (g/kg)	889.0	890.8	889.8	887.5
Crude protein (g/kg DM)	274.7	265.4	245.0	242.1
Ether extract (g/kg DM)	23.4	24.8	21.9	24.3
Crude fiber (g/kg DM)	51.0	49.1	45.0	45.4
Ash (g/kg DM)	91.5	92.5	82.5	81.7
ME (MJ/kg DM)	12.0	12.0	12.1	12.1

CG—control group; EG—experimental group; DM—dry matter; ME—metabolizable energy.

**Table 2:** The influence of humic substances on average body weight in pheasants (g/pc).

	CG	EG	SEM	*p*-Value
1st day	18.16	18.80	0.182	0.074
28th day	199.13	196.44	1.379	0.361
49th day	403.71 ^b^	415.24 ^a^	2.298	0.003

CG—control group; EG—experimental group; SEM—standard error of the mean; pc—piece. Average values in the same row marked with a superscript are statistically different (^ab^ *p* ˂ 0.05).

**Table 3:** The influence of humic substances on average daily weight gain, average daily feed intake and feed conversion ratio in pheasants.

	CG	EG	SEM	*p*-Value
Average daily weight gain (g/pc)				
1–28 days	6.46	6.34	0.050	0.258
29–49 days	9.74 ^b^	10.42 ^a^	0.149	0.011
1–49 days	7.87 ^b^	8.09 ^a^	0.045	0.004
Average daily feed intake (g/pc/day)				
1–28 days	13.25	14.38	0.337	0.097
29–49 days	35.05	35.71	0.312	0.318
1–49 days	22.60	23.52	0.253	0.062
Feed conversion ratio (kg/kg)				
1–28 days	2.05 ^b^	2.27 ^a^	0.057	0.049
29–49 days	3.60 ^a^	3.43 ^b^	0.044	0.049
1–49 days	2.87	2.91	0.028	0.564

CG—control group; EG—experimental group; SEM—standard error of the mean; pc—piece. Average values in the same row marked with different superscripts are statistically significant (^ab^ *p* ˂ 0.05).

**Table 4:** The influence of humic substances on the relative weight of digestive organs (g/100 g body weight).

	CG	EG	SEM	*p*-Value
Proventriculus	0.54	0.56	0.014	0.495
Gizzard	2.42 ^b^	2.71 ^a^	0.059	0.013
Duodenum	0.87	0.96	0.031	0.180
Jejunum	1.31 ^b^	1.54 ^a^	0.052	0.028
Ileum	1.13	1.22	0.051	0.400
Small intestine	3.32 ^b^	3.72 ^a^	0.088	0.022
Pancreas	0.34	0.33	0.012	0.728
Liver	2.36	2.43	0.052	0.533

CG—control group; EG—experimental group; SEM—standard error of the mean. Average values in the same row marked with a superscript are statistically different (^ab^ *p* ˂ 0.05).

**Table 5:** The influence of humic substances on the relative length of small intestine segments and their percentage share from the total length of the small intestine.

	CG	EG	SEM	*p*-Value
the relative length (cm/100 g body weight)				
Duodenum	3.66 ^b^	4.16 ^a^	0.095	0.008
Jejunum	8.92	9.22	0.206	0.478
Ileum	9.11	9.32	0.217	0.638
Small intestine	21.69	22.70	0.414	0.237
% of the length of the small intestine				
Duodenum	16.94 ^b^	18.35 ^a^	0.358	0.046
Jejunum	41.10	40.64	0.555	0.690
Ileum	41.96	41.01	0.501	0.356

CG—control group; EG—experimental group; SEM—standard error of the mean. Average values in the same row marked with a superscript are statistically different (^ab^ *p* ˂ 0.05).

**Table 6:** The influence of humic substances on the concentration of short-chain fatty acids (µmol/g) and pH in the contents of the cecum.

	CG	EG	SEM	*p*-Value
Acetic acid	64.17	70.33	3.520	0.398
Propionic acid	32.11	34.69	2.402	0.610
Butyric acid	18.75	19.80	1.815	0.784
Total SCFAs	115.02	124.83	6.466	0.467
Cecal pH	6.07	6.07	0.108	0.988

CG—control group; EG—experimental group; SEM—standard error of the mean; SCFAs—short-chain fatty acids.

**Table 7:** The influence of humic substances on some blood serum biochemical parameters.

	CG	EG	SEM	*p*-Value
Total protein (g/L)	35.74	33.27	0.677	0.061
Albumin (g/L)	15.30	13.93	0.395	0.128
Globulin (g/L)	20.45	19.34	0.421	0.146
Urea (mmol/L)	0.90	0.77	0.058	0.258
Uric acid (μmol/L)	455.73	387.80	21.878	0.124
Glucose (mmol/L)	19.18	20.06	0.290	0.124
Total lipids (g/L)	3.55	3.18	0.143	0.197
Cholesterol (mmol/L)	3.04	2.95	0.134	0.787
Triacylglycerols (mmol/L)	1.02	1.12	0.025	0.063
ALP (μkat/L)	33.50	31.13	1.055	0.255
AST (μkat/L)	3.40	3.30	0.201	0.778
Calcium (mmol/L)	2.38 ^a^	2.23 ^b^	0.037	0.038
Phosphorus (mmol/L)	2.21	2.06	0.056	0.180

CG—control group; EG—experimental group; SEM—standard error of the mean; ALP—alkaline phosphatase; AST—aspartate aminotransferase. Average values in the same row marked with a superscript are statistically different (^ab^ *p* ˂ 0.05).

## Data Availability

The data that support the findings of this study are available from the corresponding author upon reasonable request.
